# Prognostic significance of p27 in colorectal cancer: a meta-analysis and bioinformatics analysis

**DOI:** 10.3389/fonc.2024.1495476

**Published:** 2024-12-23

**Authors:** Jing Zou, Dong Wang, Gaoping Yin, Kexiang Lu, Kaibin Chang, He Li

**Affiliations:** ^1^ Department of Radiology, Yantai Affiliated Hospital of Binzhou Medical University, Yantai, Shandong, China; ^2^ Department of Stomach and Intestine, Yantai Affiliated Hospital of Binzhou Medical University, Yantai, Shandong, China

**Keywords:** p27, colorectal cancer, prognosis, meta-analysis, bioinformatics analysis

## Abstract

**Background:**

In the past, numerous investigations have delved into the influence of p27 (p27kip) on the prognosis and clinicopathological characteristics of colorectal cancer (CRC), yielding conclusions that are not universally statistically significant, thus rendering the discourse rather contentious.

**Methods:**

We collected available articles published before August 2024 and extracted data to analyze the association between the expression of p27 and the prognosis and clinicopathological features of CRC. In addition, we used Gene Expression Profiling Interactive Analysis (GEPIA), University of Alabama at Birmingham’s Cancer Data Analysis Portal (UALCAN), and the Human Protein Atlas (HPA) to validate our results.

**Results:**

Through an extensive examination of four prominent databases, a total of 21 original articles encompassing a cohort of 3,378 patients were identified. The findings indicated that a low expression of p27 could lead to shorter overall survival (OS) [hazard ratio (HR) = 0.44, 95% confidence interval (95%CI) = 0.31–0.61, *Z* = 4.89, *p* = 0.000] and disease-free survival (DFS) (HR = 0.40, 95%CI = 0.28–0.59, *Z* = 4.75, *p* = 0.000). In addition, a low expression of p27 predisposed tumors to the right colon [odds ratio (OR) = 0.61, 95%CI = 0.46–0.82, *Z* = 3.32, *p* = 0.001] and limited tumor differentiation (OR = 0.56, 95%CI = 0.41–0.77, *Z* = 3.62, *p* = 0.000), but had no effect on TNM staging (OR = 0.80, 95%CI = 0.52–1.22, *Z* = 1.05, *p* = 0.295), lymph node metastasis (OR = 0.90, 95%CI = 0.25–3.28, *Z* = 0.16, *p* = 0.876), and tumor size (OR = 0.94, 95%CI = 0.54–1.65, *Z* = 0.21, *p* = 0.835). The results from GEPIA and UALCAN showed that p27 had no effect on TNM staging, lymph node metastasis, DFS, and OS; moreover, there was no expression difference between tumor tissues and normal tissues. The findings from the HPA indicated that there was lower expression of p27 in tumor tissues compared with normal tissues.

**Conclusion:**

Although inconsistent results were reached with the bioinformatics analysis from this meta-analysis, it was confirmed that a low expression of p27 can adversely affect the prognosis of patients with CRC and make a meaningful impact on a part of the clinicopathological features in the meta-analysis with abundant data. In the future, predicting the prognosis of patients with CRC and guiding treatment might emerge as a significant objective.

## 1 Introduction

In 2022, colorectal cancer (CRC) accounted for over 1.9 million new cases and 904,000 deaths, positioning it as having the third highest incidence and the second highest mortality rate among 36 cancers globally across 185 countries (1). However, early CRC has a good prognosis, with no obvious symptoms, leading to a sizeable proportion of the population being diagnosed in the middle and late stages. Approximately 50% of CRC develop metastases during the disease process, and most patients with metastatic CRC cannot be cured (2). Surgery, adjuvant chemoradiotherapy, and immunotherapy are the main treatments for CRC. Nevertheless, the 5-year survival rate for metastatic CRC stands at a mere 14% (3), which remains fairly inadequate. Currently, CRC poses a significant threat to human life and health, presenting considerable difficulties and challenges in clinical diagnosis and treatment. This underscores the necessity of enhancing the prognosis for CRC. The clinical application of tumor markers has provided great convenience for the disease assessment of CRC. However, these tumor markers sometimes lack the desired specificity and sensitivity, which requires strengthening their exploration to improve the treatment of CRC. The intricate molecular mechanism governing cell cycle regulation encompasses the interplay between cyclins, cyclin-dependent kinases (CDKs), and cyclin-dependent kinase inhibitors (CDKIs). CDKIs consist of two families—CIP/KIP (p27Kip1, p21Cip1, and p57Kip2) and INK4 (p15Ink4b, p16Ink4a, p18Ink4c, and p19Ink4d)—which serve to inhibit the activity of CDK. p27kip, also named p27, a member of the KIP family (4), is encoded by the *CDKN1B* gene located on chromosome 12p13 (5, 6). As an important regulator of the cell cycle, p27 could prevent the G1/S transition by inhibiting CDK2–cyclin E and CDK4–cyclin D (7). Once p27 is absent in the G1 phase, the cell cycle will more easily transition from the G1 to the S phase, which could encourage cell division (8).

At present, many clinical studies and database data have elaborated on the association between p27 and CRC. However, the data in these clinical studies have the disadvantages of being from small sample sizes and discrete results; thus far, they have not been systematically collated. This study will summarize these clinical data through a meta-analysis and combine these with the results of bioinformatics analysis to further determine whether p27 can be a target for prognosis assessment and treatment in CRC.

## 2 Materials and methods

### 2.1 Search strategy

Two scholars employed pertinent terminology to explore articles published in PubMed, Embase, Cochrane Library, and Web of Science from the inception of the database up to August 2024. The pertinent terminology included: “cyclin-dependent kinase inhibitor,” “cdk inhibitor,” “CDKN1B,” “p27kip1,” “p27,” “colorectal neoplasms,” “colorectal neoplasm,” “colorectal tumors,” “colorectal tumor,” “colorectal cancer,” “colorectal cancers,” “colorectal carcinoma,” and “colorectal carcinomas.”

### 2.2 Inclusion and exclusion criteria

The following inclusion criteria were established for original articles: 1) the patient was pathologically diagnosed with CRC; 2) the language is limited to English; 3) the expression of p27 was evaluated by immunohistochemistry (IHC); and 4) prognostic or clinicopathological data were provided. The exclusion criteria were: 1) meta-analyses, reviews, papers, letters, or case reports; 2) studies involving non-human tissues; 3) insufficient data to compute the hazard ratio (HR), odds ratio (OR), and their 95% confidence interval (95%CI); and 4) Newcastle-Ottawa Scale (NOS) score <6 (9).

### 2.3 Data extraction and quality assessment

Two researchers extracted data from the articles, including the first author, year of publication, country, age, sample size, staging, male-to-female ratio, cut-off value, and outcome indicators. All original articles were scored using the NOS. The scoring system is designed to vary between 0 and 9. Any disputes arising during this process were addressed through a discussion involving a third researcher.

### 2.4 Statistical analysis

This meta-analysis was conducted by state15 (StataCorp, College Station, TX, USA). A *p <* 0.05 was considered statistically significant. HR, with its 95%CI, was used to evaluate the effect of p27 on overall survival (OS) and disease-free survival (DFS). If the original study did not provide survival data, these were calculated using the Kaplan–Meier curve. OR was used to evaluate the effect of p27 on the clinicopathological features. The chi-square-based *Q* test and *I* ^2^ test were used to evaluate heterogeneity. When the heterogeneity was high (*p* < 0.05, *I* ^2^ > 50%), a random effects model was used. When the heterogeneity was low (*p* > 0.05, *I* ^2^ < 50%), a fixed effects model was used. Begg’s test was used to evaluate publication bias. A *p* < 0.05 suggests the presence of publication bias. The stability of the results was assessed through sensitivity analysis.

### 2.5 Bioinformatics analysis

Utilizing the data sourced from The Cancer Genome Atlas (TCGA), the University of Alabama at Birmingham’s Cancer Data Analysis portal (UALCAN) was employed to examine the effect of p27 on lymph node metastasis, TNM staging, and OS. Utilizing the data sourced from TCGA and the Genotype-Tissue Expression Project (GTEx), Gene Expression Profiling Interactive Analysis (GEPIA) was employed to examine the effect of p27 on TNM staging, DFS, and OS. A *p* < 0.05 was considered statistically significant.

The Human Protein Atlas (HPA) database was employed to examine the protein expression levels of p27 in normal tissue and in CRC tissue. The initial score from quantity was graded as follows: 0, none; 1, <25%; 2, 25–75%; and 3, >75%. The initial score from intensity was graded as follows: 0, negative; 1, weak; 2, moderate; and 3, strong. The final score was determined by multiplying the scores above. The expression level was determined as high or low expression using a cutoff value. According to the average score, 3.69 was regarded as the cutoff. Fisher’s exact test was used to evaluate the expression differences between normal tissue and tumor tissue. A *p* < 0.05 was considered statistically significant.

## 3 Results

### 3.1 Study characteristics

**Figure 1:**
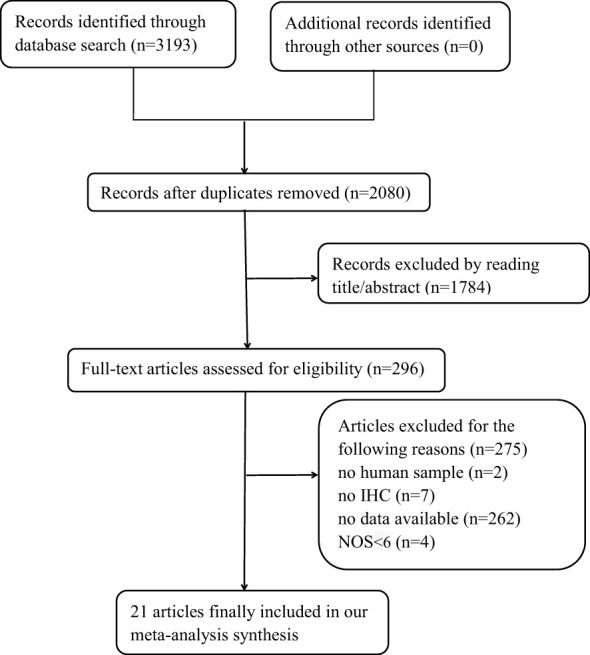
Flowchart of article screening.

**Figure 1** shows that 3,193 records were sourced from the PubMed, Cochrane Library, Web of Science, and Embase databases, with 1,113 being excluded due to duplication. After a review of the titles and abstracts of the remaining records, it was determined that 1,784 records be excluded due to a lack of alignment with the thematic focus of the study. After reading the full text of the remaining records, 275 articles were excluded for the following reasons: 1) no human sample; 2) no IHC; 3) no available data; and 4) with NOS <6. A total of 21 original articles (10–30) included in our meta-analysis were published from 1997 to 2017 and included a total of 3,378 patients with CRC. Of these articles, 5 were from Asia, 12 from Europe, and 4 from the USA. These original articles are high-quality studies, with NOS scores ranging from 6 to 9 (**Table 1**).

**Table 1:** Basic features of the original studies included.

Study	Year	Country	Age (years)	Sample (men/women)	Stage^a^	Cutoff	Proportion^b^	Outcome	NOS
Loda et al. (10)	1997	USA		149	I–IV	50%	15/149	OS	6
Ciaparrone et al. (11)	1998	Italy	67.74 (32–92)	40 (22/18)	A–D	50%	11/40		8
Belluco et al. (12)	1999	Italy	64 (34–92)	124 (72/52)	I–III		17/124	OS, DFS	8
Palmqvist et al. (13)	1999	Sweden		89 (48/41)	A–C	50%	39/89		7
Yao et al. (14)	2000	Singapore		127 (78/49)	I–IV		28/127	OS	8
Zhang and Sun (15)	2001	Sweden		178 (100/78)	A–D	10%	91/108	OS	7
Rossi et al. (16)	2002	USA	75 (30–101)	187 (91/96)	I–III	25%	104/155	OS, DFS	6
Noguchi et al. (17)	2003	Japan	64	80		30%	37/80	OS	6
Prall et al. (18)	2004	Germany	64.8 (29–90)	184 (93/91)	I–IV	50%	34/164	OS	9
Rosati et al. (19)	2004	Italy	66 (29–79)	103 (79/24)	B, C	10%	28/103	OS, DFS	6
Wu et al. (20)	2005	USA	65.2	168 (88/80)	II–III	50%	77/168	OS	8
Shapira et al. (21)	2005	Israel		80 (44/36)	I–IV	50%	44/80	OS	6
Sarli et al. (22)	2006	Italy	70.1 (41–94)	108 (55/53)	I–IV	50%	33/108		8
Li et al. (23)	2007	China	60 (22–82)	127 (61/66)	I–III		48/127	DFS	6
Zlobec et al. (24)	2007	Switzerland		587				OS	6
Ioachim (25)	2008	Greece	64.92 (26–86)	97	B, C	50%	80/97		8
Leopoldo et al. (26)	2008	Italy		55	I–IV	50%	32/55		8
Ogino et al. (27)	2009	USA	66.4	630 (268/362)	I–IV	20%	170/300	OS	7
Bottini et al. (28)	2009	Italy	71 (40–94)	70 (30/40)			34/70		9
Al-Maghrabi et al. (29)	2011	Saudi Arabia	58 (24–90)	65 (32/33)	I–IV	10%	30/65		8
Bochis et al. (30)	2017	Romania		130 (70/60)	I–IV	50%	55/88	OS, DFS	6

*OS*, overall survival; *DFS*, disease-free survival.

a TNM or Dukes.

b Proportion of p27 low expression in the total sample size.

### 3.2 Association between p27 expression and OS

**Figure 2:**
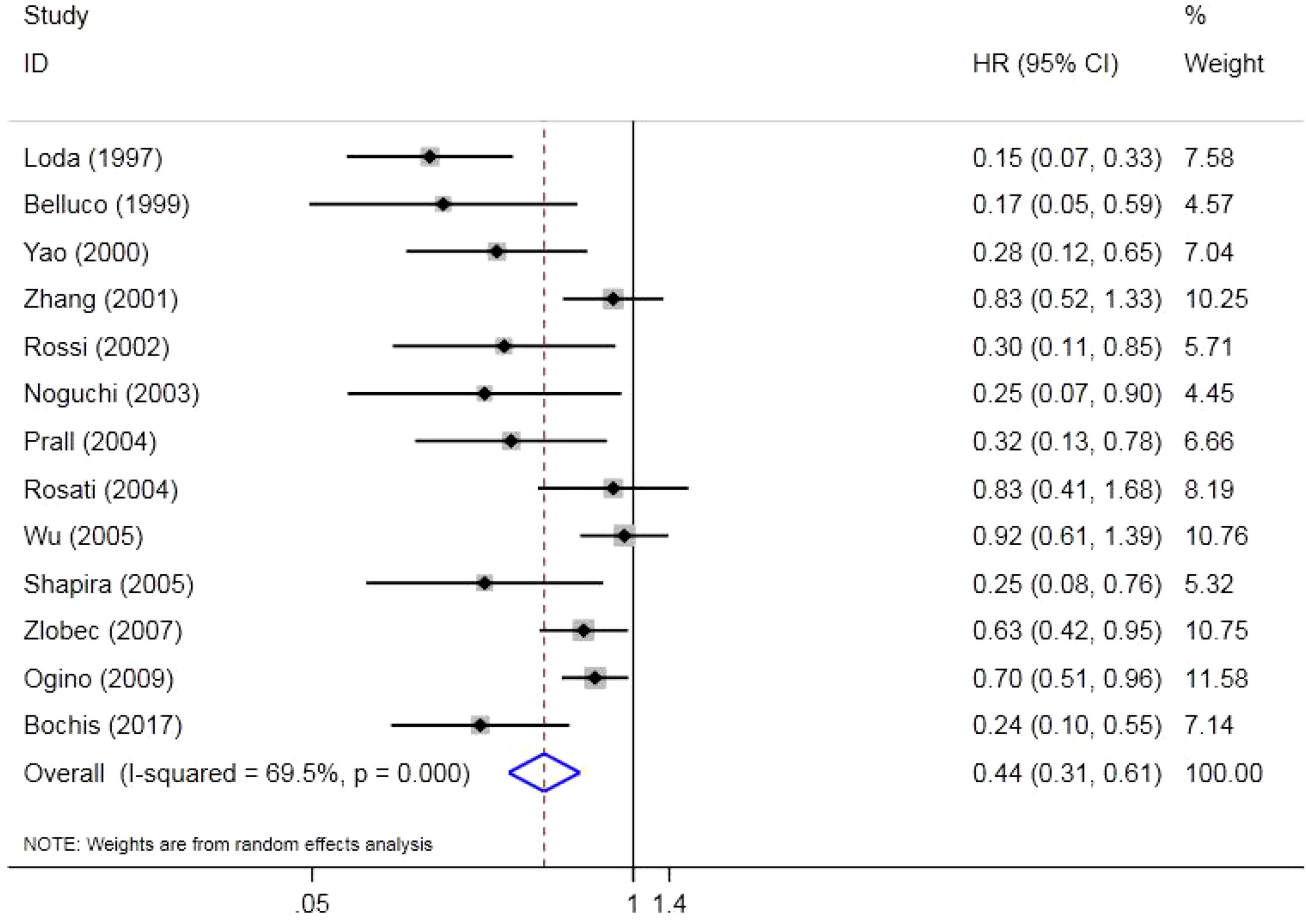
Forest plot of the association between p27 expression and overall survival (OS).

There were 13 original studies including 2,523 individuals that investigated the effect of p27 on OS. The results indicated that a low expression of p27 could shorten OS in CRC (HR = 0.44, 95%CI = 0.31–0.61, *Z* = 4.89, *p* = 0.000). Due to the high heterogeneity (*p* = 0.000, *I* ^2^ = 69.5%), a random effects model was used (**Figure 2**).

### 3.3 Association between p27 expression and DFS

**Figure 3:**
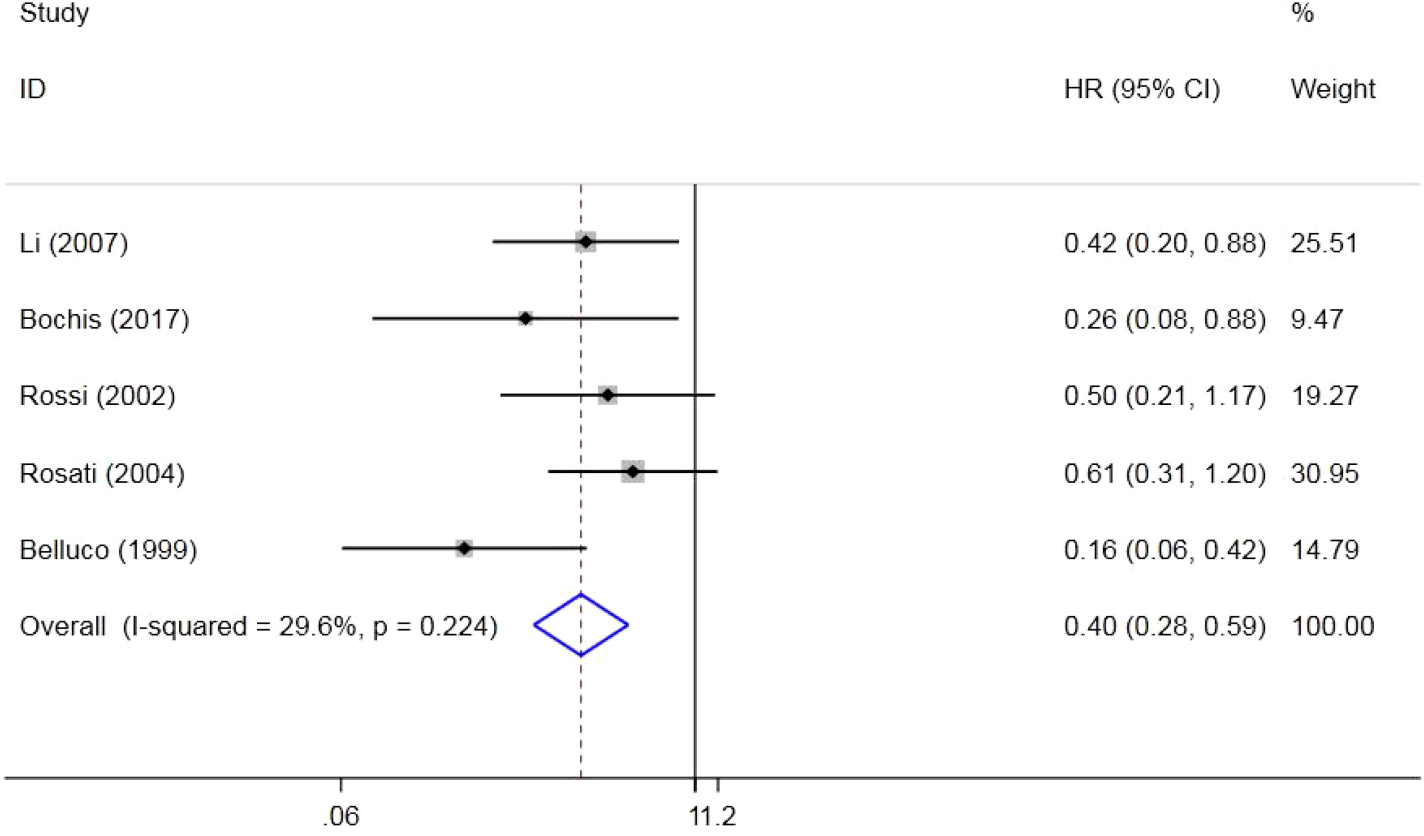
Forest plot of the association between p27 expression and disease-free survival (DFS).

Five original studies that included 597 individuals investigated the effect of p27 on DFS. The results indicated that a low expression of p27 could shorten the DFS of CRC (HR = 0.40, 95%CI = 0.28–0.59, *Z* = 4.75, *p* = 0.000). Due to the low heterogeneity (*p* = 0.224, *I* ^2^ = 29.6%), a fixed effects model was used (**Figure 3**).

### 3.4 Subgroup analysis

A detailed subgroup analysis was conducted considering factors such as the study region, publication year, sample size, and cutoff values. The findings indicated that the effect of p27 on OS was statistically significant across all subgroups (**Table 2**).

**Table 2:** Subgroup analysis of the association between p27 expression and overall survival (OS).

Subgroup analysis	Study	Model	HR (95%CI)	*p*	Heterogeneity	
					*I* ^2^	*p*
Region						
Asia	3	Fixed	0.26 (0.15–0.48)	0	0%	0.983
Other	10	Random	0.49 (0.34–0.69)	0	72%	0.000
Year						
<2004	6	Random	0.29 (0.15–0.58)	0	73.3%	0.002
≥2004	7	Random	0.58 (0.41–0.80)	0.001	57.8%	0.027
Sample						
>100	11	Random	0.46 (0.33–0.66)	0	71.7%	0.000
<100	2	Fixed	0.25 (0.11–0.58)	0.001	0	1
Cutoff						
≥50%	5	Random	0.32 (0.14-0.71)	0.005	82.8%	0.000
<50%	5	Fixed	0.69 (0.55–0.87)	0.002	30.7%	0.217

### 3.5 Sensitivity and publication analyses

Sensitivity analysis was conducted on OS (**Supplementary Table S1**) and DFS (**Supplementary Table S2**). The results showed that regardless of which original study was removed, there was no significant change in the final effect value. Begg’s test was employed to assess publication bias concerning OS (**Supplementary Figure S1**) and DFS (**Supplementary Figure S2**). The findings indicated an absence of publication bias in terms of OS (*p* = 0.1) and DFS (*p* = 0.221).

### 3.6 Association between p27 expression and clinicopathological features

Our meta-analysis showed that a low expression of p27 could predispose tumors to the right half colon (left *vs*. right; *n* = 9, OR = 0.61, 95%CI = 0.46–0.82, *Z* = 3.32, *p* = 0.001) (**Supplementary Figure S3A**) and inhibit tumor differentiation (high, medium *vs*. low; *n* = 12, OR = 0.56, 95%CI = 0.41–0.77, *Z* = 3.62, *p* = 0.000) (**Supplementary Figure S3B**). However, it had no effect on TNM staging (I, II *vs*. IIII, IV; *n* = 8, OR = 0.80, 95%CI = 0.52–1.22, *Z* = 1.05, *p* = 0.295) (**Supplementary Figure S3C**), lymph node metastasis (no *vs*. yes; *n* = 3, OR = 0.90, 95%CI = 0.25–3.28, *Z* = 0.16, *p* = 0.876) (**Supplementary Figure S3D**), and tumor size (≤5 cm *vs*. >5 cm; *n* = 2, OR = 0.94, 95%CI = 0.54–1.65, *Z* = 0.21, *p* = 0.835) (**Supplementary Figure S3E**).

### 3.7 Results of the bioinformatics analysis

**Figure 4:**
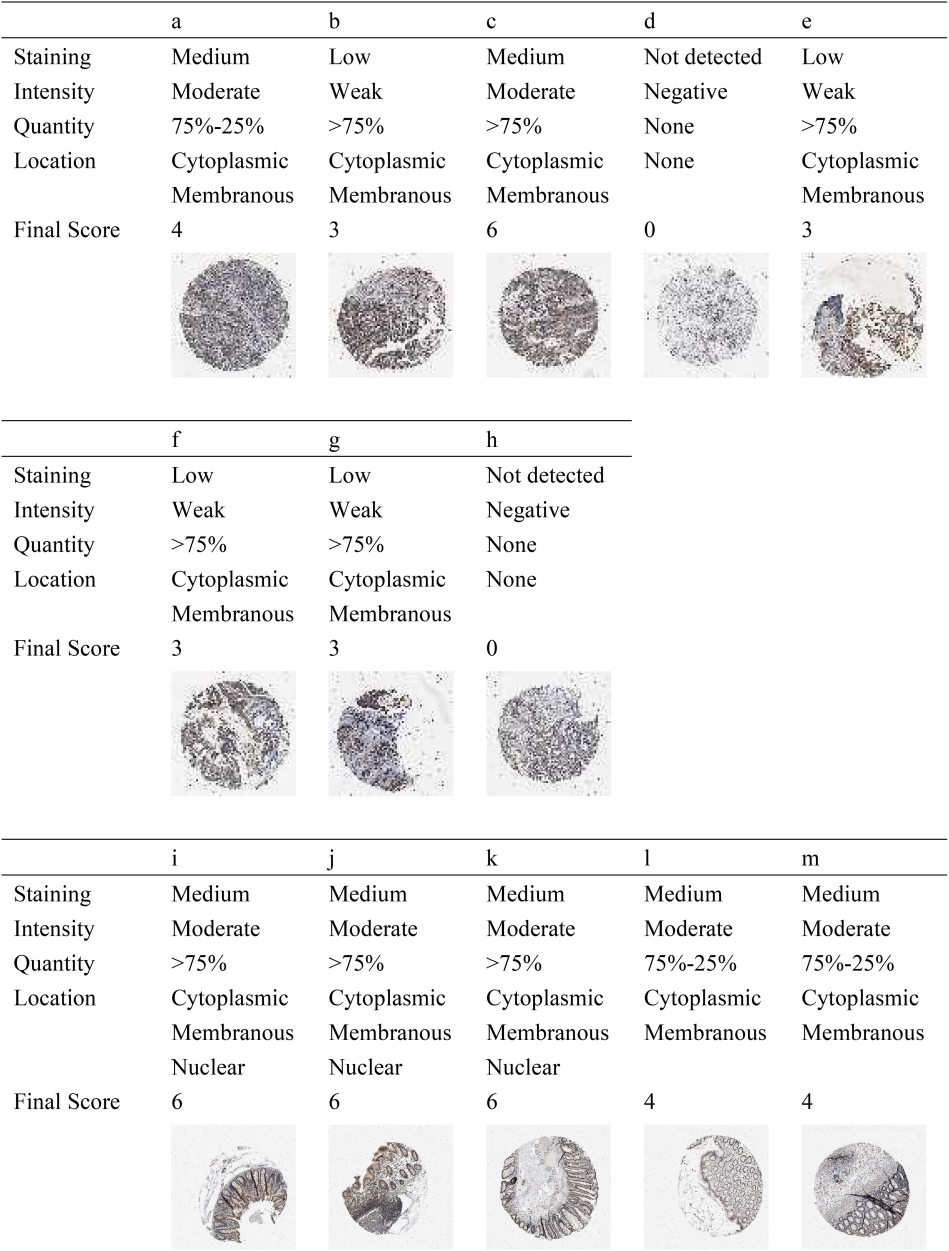
Protein expression level of p27 in colorectal cancer (CRC) tissues **(A–H)** and in normal tissues **(I–M)** using the Human Protein Atlas (HPA).

As shown in **Figure 4**, there were five normal colorectal samples and eight CRC samples in HPA. Combining the strength, area, and quantity, we found a trend of a lower expression of p27 in tumor tissues compared with normal tissues. According to the cutoff, there was a 100% (5/5) high expression rate in normal tissues and a 25% (2/8) high expression rate in tumor tissues. Further statistical verification showed that the expression of p27 in normal tissues was higher than that in tumor tissues (*p* = 0.02098). In the assessment of the expression location, no p27 expression was found in the nucleus of tumor tissue cells compared with normal tissue cells.

As per the findings from GEPIA, the box plot indicated that there were no differences in the expression of *CDKN1B* messenger RNA (mRNA) between normal and tumor tissues in colon cancer (*p* > 0.05) (**Supplementary Figure S4A**) and rectal cancer (*p* > 0.05) (**Supplementary Figure S5A**). According to the pathological stage plot, although the expression level of *CDKN1B* mRNA slightly differed in the four stages, it was not significantly associated with the TNM staging of either colon (*p* > 0.05) (**Supplementary Figure S4B**) or rectal cancer (*p* > 0.05) (**Supplementary Figure S5B**). Kaplan–Meier analysis with the log-rank test based on 270 patients with colon cancer indicated that the expression level of *CDKN1B* mRNA had no significant effect on OS (*p* > 0.05) (**Supplementary Figure S4C**) or DFS (*p* > 0.05) (**Supplementary Figure S4D**). Kaplan–Meier analysis with the log-rank test based on 92 patients with rectal cancer indicated that the expression level of *CDKN1B* mRNA had no significant effect on OS (*p* > 0.05) (**Supplementary Figure S5C**) or DFS (*p* > 0.05) (**Supplementary Figure S5D**).

According to the findings from UALCAN, the box plot based on 283 patients with colon cancer (*p* > 0.05) (**Supplementary Figure S6A**) and 162 patients with rectal cancer (*p* > 0.05) (**Supplementary Figure S7A**) indicated that there was no significant correlation between the expression level of *CDKN1B* mRNA and lymph node metastasis. In colon cancer, the box plot based on 274 patients clearly indicated that there was no significant correlation between the expression level of *CDKN1B* mRNA and TNM staging (*p* > 0.05) (**Supplementary Figure S6B**). In rectal cancer, the box plot based on 156 patients indicated that, although the expression level of *CDKN1B* mRNA slightly differed in the different stages, it had no significant effect on TNM staging (*p* > 0.05) (**Supplementary Figure S7B**). The survival curve based on 209 patients with low/medium expression and 70 patients with high expression indicated that the expression level of *CDKN1B* mRNA had no significant effect on OS in colon cancer (*p* > 0.05) (**Supplementary Figure S6C**). The survival curve based on 124 patients with low/medium expression and 41 patients with high expression indicated that the expression level of *CDKN1B* mRNA had no significant effect on OS in rectal cancer (*p* > 0.05) (**Supplementary Figure S7C**).

## 4 Discussion

The emergence and progression of intestinal carcinoma is an extensive, multistage process characterized by alterations in numerous genes, encompassing a complex interplay of genetic regulation and multifactorial influences (31).

As an important component of the CIP/KIP family in CDKIs, p27 belongs to the intrinsically unstructured proteins (IUPs) that lack a stable secondary/tertiary structure and can only fold when interacting with a substrate, a property that strongly enhances its activity spectrum (32, 33). p27 inhibits the activity of cyclin/CDK via the N-terminal, which contains a highly conserved region known as the “kinase inhibitory domain (KID)” (34, 35). The KID consists of three sub-domains: the cyclin binding sub-domain (D1), the CDK binding sub-domain (D2), and the linker sub-domain (LH) that joins D1 and D2 (35). D1 and D2 undergo a conformational change upon the binding of p27 to cyclins and CDKs. Nevertheless, LH maintains an α-helix configuration regardless of its binding to cyclin–CDK (36). A portion of the D2 sub-domain can enter the catalytic center in the CDK to compete with ATP, which can prevent the transfer of phosphate to the substrate to inhibit CDK activity (34). Special domains in D1 can be used to block access to the last critical substrate-docking site on cyclinD and cyclinE, which allows p27 to adjust and inhibit CDK4 and CDK2 (34, 37, 38). The C-terminal domain (CTD), which is characterized as an intrinsically disordered region, possesses the ability to connect with a diverse array of proteins through various conformations and includes a nuclear localization signal (NLS) (36). The C-terminal region of p27 facilitates interactions with transcriptional regulators on chromatin, while its N-terminal associates with the cyclin–CDK complex. This connection enables p27 to phosphorylate specific targets on the chromatin after CDK activation, thereby regulating gene transcription (36).

p27 is regulated by posttranslational modifications, such as phosphorylation and acetylation. When phosphorylated, the protein level of p27 is degraded through ubiquitin-dependent proteolysis (39). As a substrate-recruiting F-box protein, S-phase kinase-associated protein-2 (SKP2) forms the SCFSKP2 ubiquitin ligase complex with cullin-1 (CUL1), RING box protein-1 (RBX1), and S-phase kinase-associated protein-1 (SKP1) (40). Phosphorylated p27 could be degraded by the SCFSKP2 ubiquitin ligase complex after being recognized by SKP2 (41). A multitude of tyrosine kinases, such as those belonging to the Src family, have the capability to phosphorylate particular tyrosine residues (Y88 and Y74) within the D2 sub-domain of p27. This phosphorylation event induces a partial activation of CDK within the trimeric complexes comprising p27, cyclin, and CDK (42–45). The partially activated CDK may facilitate the efficient phosphorylation of the Y-phosphorylated p27 at threonine 187 (T187), resulting in the recognition of p27 by SKP2. In a similar manner, the acetyl transferase p300/CBP associating factor (PCAF) acetylates p27 at K100, leading to its subsequent ubiquitination and degradation by the proteasome within the nucleus (46).

Conversely, p27 can also have a favorable effect on the activity of CDK. For example, the active CDK4 complex contains both p27 and cyclinD, and a large proportion of p27 forms complexes with CDK4–cyclinD in proliferating cells (7, 47–49). The active CDK4–cyclinD complex could not be assembled without p27 and p21 in embryonic fibroblasts (50). Simultaneously, as a form of unstructured protein, p27 can engage in various cellular activities independently of CDK, including cell motility and the activation of autophagy, which may facilitate the onset and progression of cancer (6).

Therefore, the effect of p27 on tumors is multidirectional and complex, which could easily lead to controversies for prognosis. In order to solve this issue, some scholars have used meta-analysis to elucidate the influence of p27 on the prognosis of gastric cancer (51), liver cancer (52), oral squamous cell carcinoma (53), and ovarian cancer (54). At present, the association between p27 and the prognosis of CRC still lacks final conclusions. Therefore, we conducted meta-analysis and bioinformatics analysis. In the meta-analysis, a total of 21 original studies were included, which included 3,378 patients. Using these rich data, it was concluded that the low expression of p27 can shorten the OS and DFS of patients with CRC and adversely affect tumor differentiation. In addition, a low expression of p27 can predispose tumors to more likely occur in the right colon. However, the expression of p27 had no significant effect on TNM staging, tumor size, and lymph node metastasis. The results of the subgroup analysis showed that the effect of p27 on CRC was not affected by publication year, cutoff, different regions, and sample size. At the same time, the sensitivity analysis confirmed the stability of the prognostic results, which allowed us to reach a more reliable conclusion. Publication bias was evaluated for OS and DFS using Begg’s test. No publication bias was found, which indicated that the research results are comprehensive.

In this context, we assert that the integration of mRNA with protein can yield more robust biomarkers. In HPA, it was found that the expression level of the p27 protein in normal tissues was higher than that in tumor tissues. Unlike p27 in normal tissue cells, which is distributed in the cytoplasm, membrane, and the nucleus, p27 in tumor tissue cells was not found in the nucleus. Previous studies have shown that the subcellular localization of p27 plays an important role in the regulation of cellular function. Cytoplasmic p27 promotes cell proliferation, autophagy, and cell survival, while nuclear p27 inhibits cell proliferation and promotes apoptosis, quiescence, and senescence (55–57). When isolated into the cytoplasm, p27 can promote tumor development (39, 58). It has been confirmed that the cytoplasmic dislocation of p27 is associated with poor prognosis in a variety of cancers (59, 60). Moreover, the absence of nuclear p27 is an important predictor of poor prognosis in CRC (10, 17).

The bioinformatics findings from GEPIA and UALCAN indicated that p27 did not influence lymph node metastasis or the TNM staging, thereby corroborating the results of our meta-analysis. However, the database results showed that the expression of p27 had no significance for OS and DFS and that the expression of p27 in tumor tissues was not statistically different from that in normal tissues, which is inconsistent with the results of our meta-analysis and HPA. This may be because *CDKN1B* mRNA was used in the database, while the original studies in our meta-analysis studied p27 at the protein level. Generally, the levels of mRNA expression and the associated proteins in cancer cells and tumor tissues do not consistently align with one another (61–63). Prior research examining the proteomics of colon and rectal cancer within TCGA database revealed that variations in the protein expression could not be inferred from the quantity of the mRNA transcripts (64). The abundance of mRNA is an important marker of the presence of a protein, a condition of whether a protein is detectable inside a cell. However, only 40% of the variation in the protein concentration can be explained by mRNA abundance (63, 65, 66). The protein formation and maintenance in cells need to undergo a process from the transcription, processing, and degradation of mRNA to the translation, localization, modification, and programmed destruction of the protein itself (67), which may have contributed to the difference in the abundance and structure between the protein and mRNA. The mRNA may be subject to various posttranscriptional regulations (63, 68), and these posttranscriptional regulations are not affected by the upregulation or the downregulation of the genes (69). Furthermore, the target protein is likely to engage with other proteins within the cellular environment, thereby influencing its localization and functionality (70).

Despite the inclusion of rich raw data and the use of strict statistical methods, the following limitations were difficult to avoid. Firstly, a proportion of the HRs was obtained through the survival curve, which would inevitably lead to calculation errors. Secondly, Begg’s test was used to evaluate publication bias in DFS, and the number of original articles in this study was less than 10, which may have rendered the results unreliable. Finally, most of the original articles in the meta-analysis targeted CRC, while the database provided data on colon or rectal cancer, which made determining a clear contrast difficult.

## 5 Conclusions

According to the meta-analysis and the bioinformatics analysis, the following conclusions were drawn. At the mRNA level, p27 had no effect on lymph node metastasis, staging, and prognosis. At the protein level, p27 had a lower expression in tumor tissues and adversely affected prognosis and differentiation, as well as predisposed tumors to the right colon. In the future, p27 could serve as a significant tumor marker to help address the challenges associated with CRC.
