# Supplementary material for: Prognostic significance of p27 in colorectal cancer: a meta-analysis and bioinformatics analysis
Source: Front Oncol. 2024 Dec 23;14:1495476. doi: 10.3389/fonc.2024.1495476 (PMC11751620; doi:10.3389/fonc.2024.1495476)
Supplement: Supplementary file 1 [file DataSheet1.doc]

**Prognostic significance of p27 in colorectal cancer: a meta-analysis and bioinformatics analysis**

**Supplementary Figures and Tables**

**Jing Zou^1#^, Dong Wang^2#^, Gaoping Yin^2#^, Kexiang Lu^2^, Kaibin Chang^2^,** **He Li2*[[1]](#footnote-2)**

**Table. S1** Sensitivity analysis of the association between p27 expression and OS

| Article omitted | Estimate HR | Lower(95%CI) | Upper(95%CI) |
| --- | --- | --- | --- |
| Loda (1997) | 0.49691118 | 0.36807055 | 0.67085161 |
| Belluco(1999) | 0.45889273 | 0.32934996 | 0.63938838 |
| Yao (2000) | 0.45147691 | 0.32044165 | 0.63609521 |
| Zhang(2001) | 0.40139924 | 0.27939922 | 0.57667072 |
| Rossi (2002) | 0.44514263 | 0.3154768 | 0.62810312 |
| Noguchi(2003) | 0.44724251 | 0.31825804 | 0.62850215 |
| Prall (2004) | 0.4446405 | 0.31436325 | 0.62890676 |
| Rosati(2004) | 0.40861299 | 0.28642492 | 0.58292615 |
| Wu (2005) | 0.3981267 | 0.27984168 | 0.56640908 |
| Shapira(2005) | 0.45020787 | 0.32037732 | 0.63265129 |
| Zlobec(2007) | 0.40829824 | 0.27931467 | 0.59684461 |
| Ogino(2009) | 0.40048874 | 0.27219967 | 0.58924108 |
| Bochis (2017) | 0.45968534 | 0.32837646 | 0.64350109 |
| Combined | 0.43574503 | 0.31238631 | 0.60781708 |

**Table. S2** Sensitivity analysis of the association between p27 expression and DFS

| Article omitted | Estimate HR | Lower(95%CI) | Upper(95%CI) |
| --- | --- | --- | --- |
| Belluco(1999) | 0.47434486 | 0.31626629 | 0.71143543 |
| Rossi (2002) | 0.3838512 | 0.25310589 | 0.5821348 |
| Rosati(2004) | 0.33575882 | 0.21402553 | 0.52673148 |
| Li (2007) | 0.3985453 | 0.25833992 | 0.61484248 |
| Bochis(2017) | 0.42296844 | 0.28543991 | 0.62675994 |
| Combined | 0.4039126 | 0.27783397 | 0.5872046 |

**Fig. S1** Begg’s test of the association between p27 expression and OS

**Fig.S2** Begg’s test of the association between p27 expression and DFS

| a | b |
| --- | --- |
| 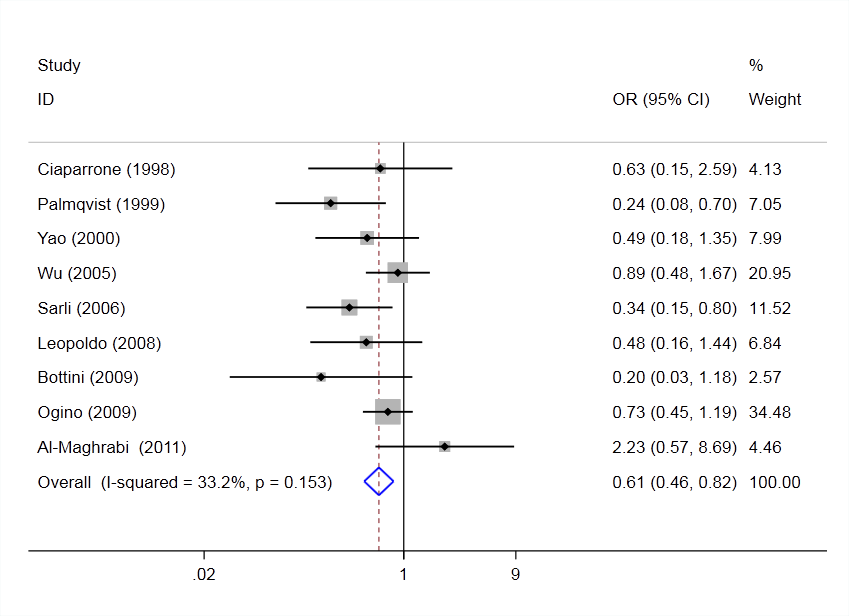 | 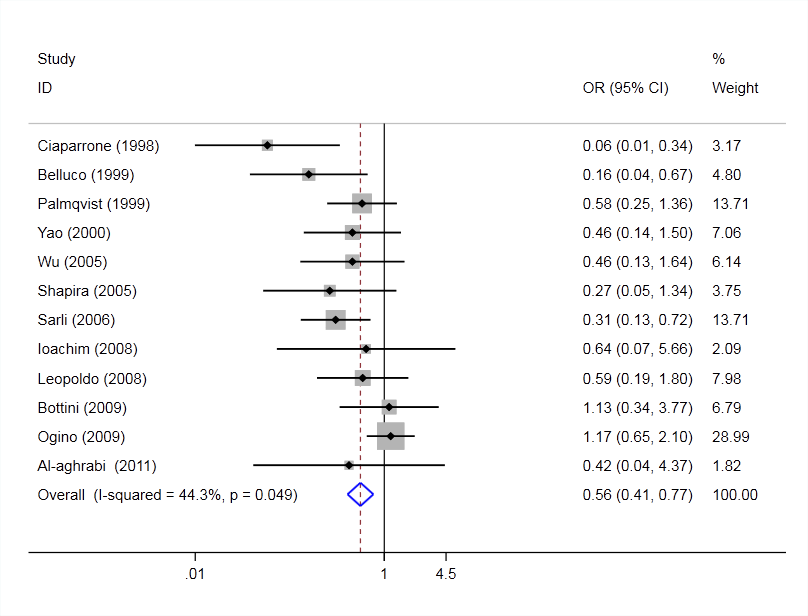 |

| c | d |
| --- | --- |
| 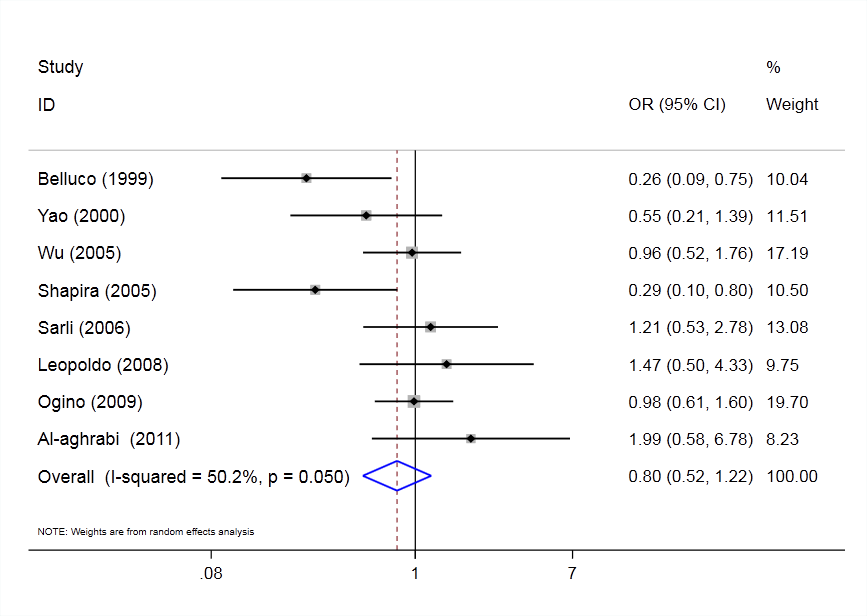 | 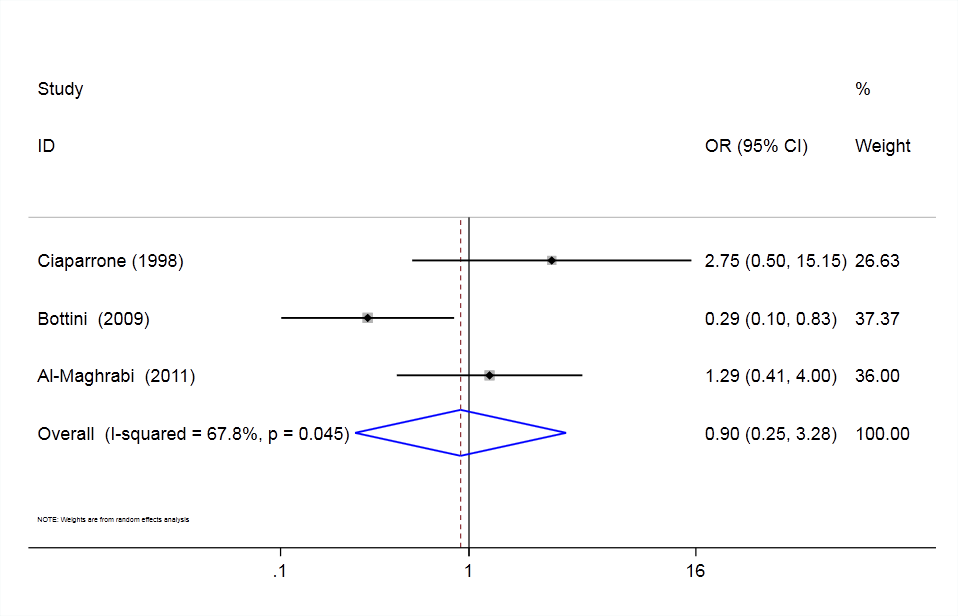 |

| e |
| --- |
| 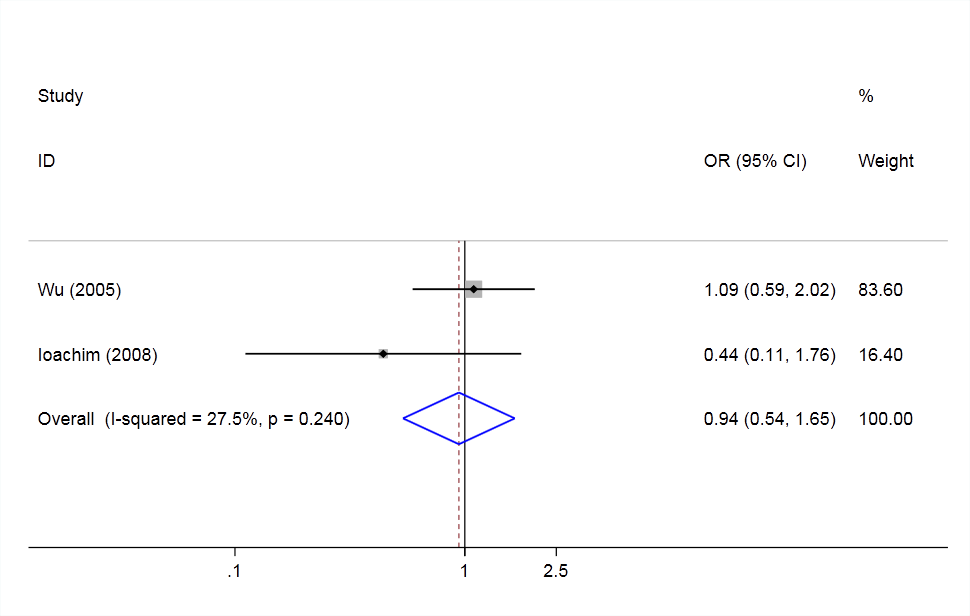 |

**Fig. S3** Forest plot of the association between p27 expression and clinicopathological features: tumor location(a), tumor differentiation(b), TNM staging(c), [lymph node](javascript:;) metastasis(d), tumor size(e).

| a | b |
| --- | --- |
|  | 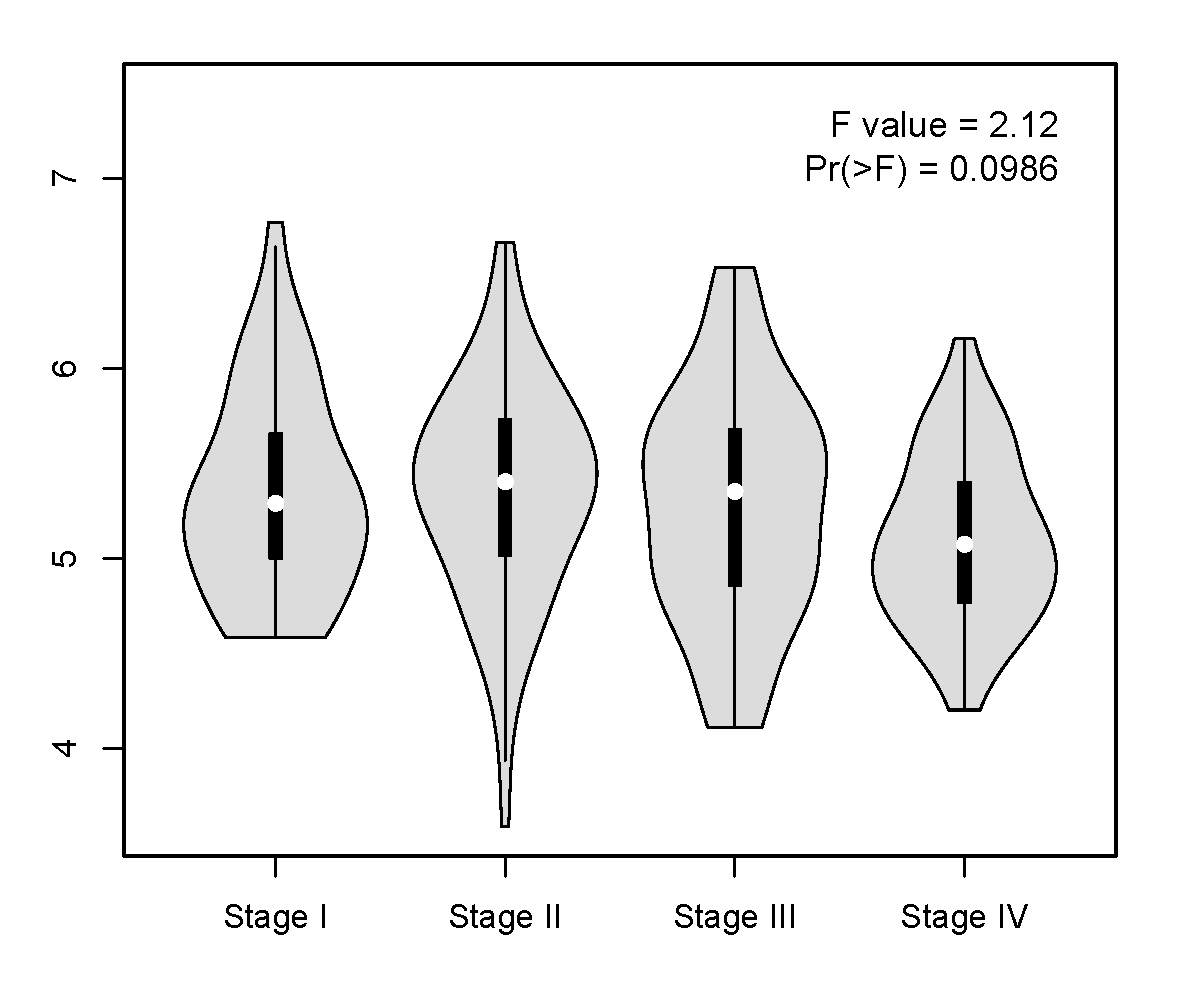 |
| c | d |
| 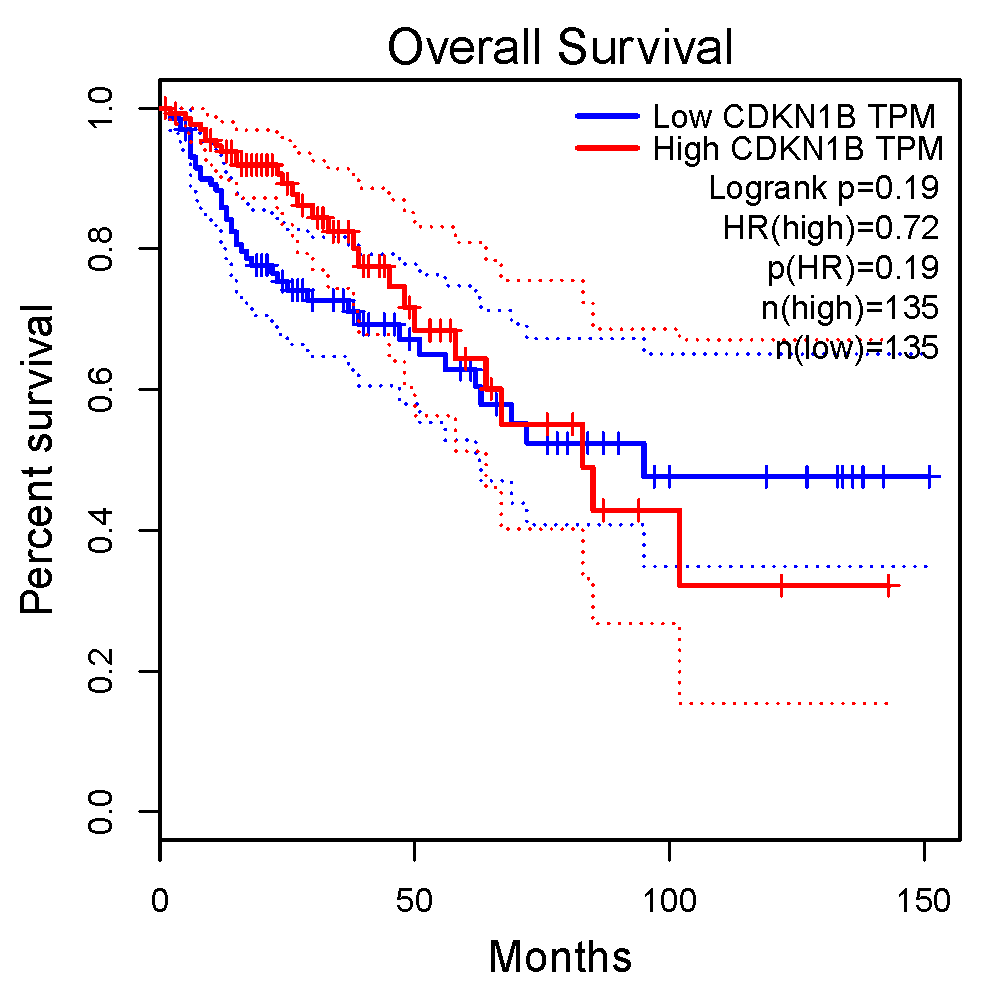 | 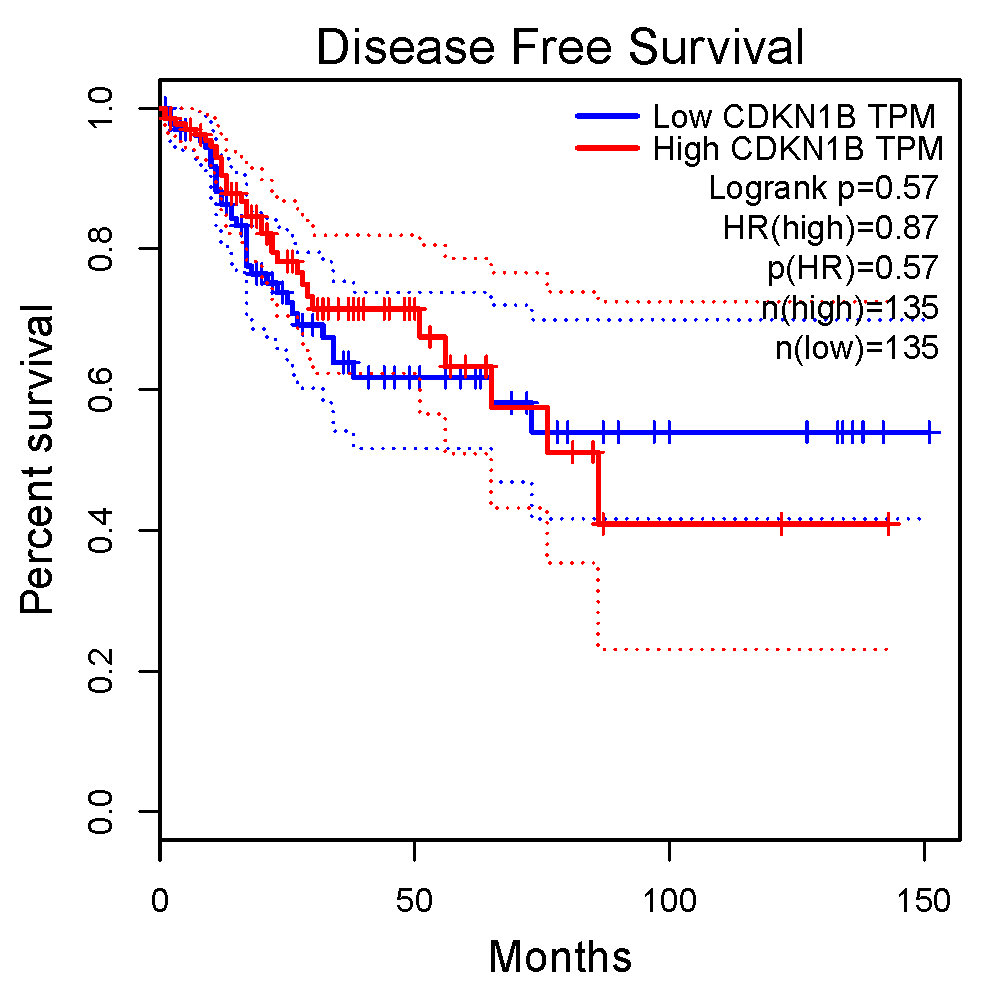 |

**Fig. S4**  Expression and clinical significance of CDKN1B mRNA in colon cancer according to GEPIA: a. the expression difference of CDKN1B mRNA in cancer tissues and normal tissues; b. the association between CDKN1B mRNA expression and TNM staging; c. the association between CDKN1B mRNA expression and OS; d. the association between CDKN1B mRNA expression and DFS.

| a | b |
| --- | --- |
| 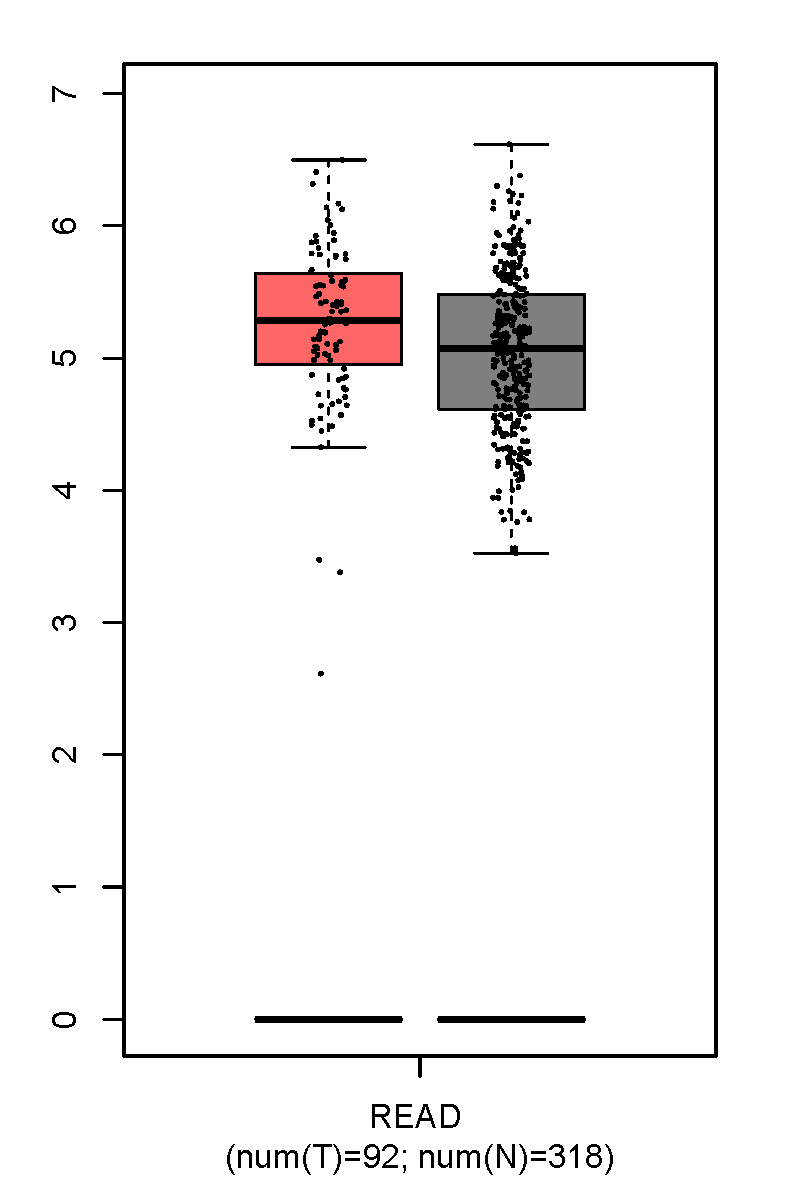 | 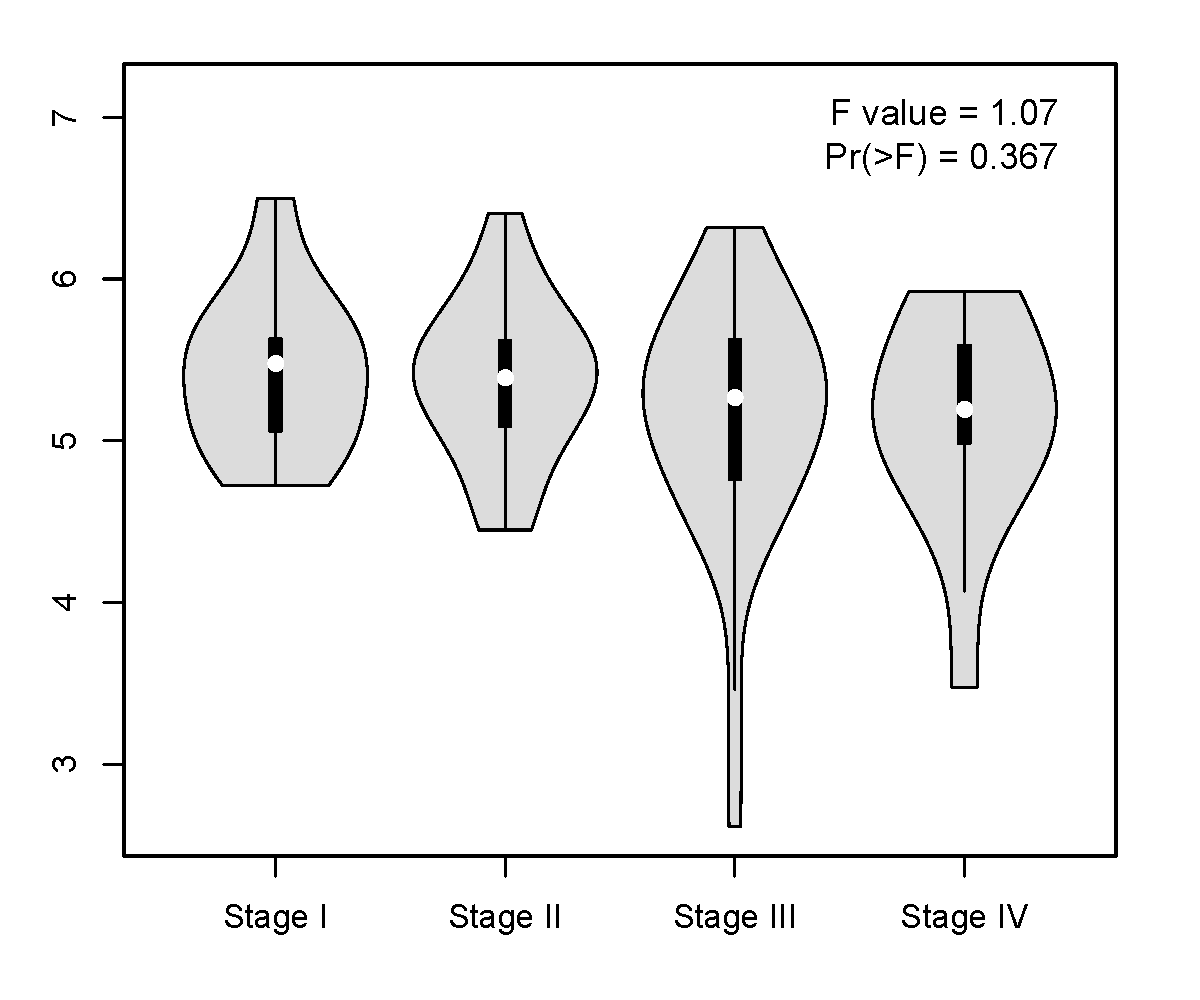 |
| c | d |
| 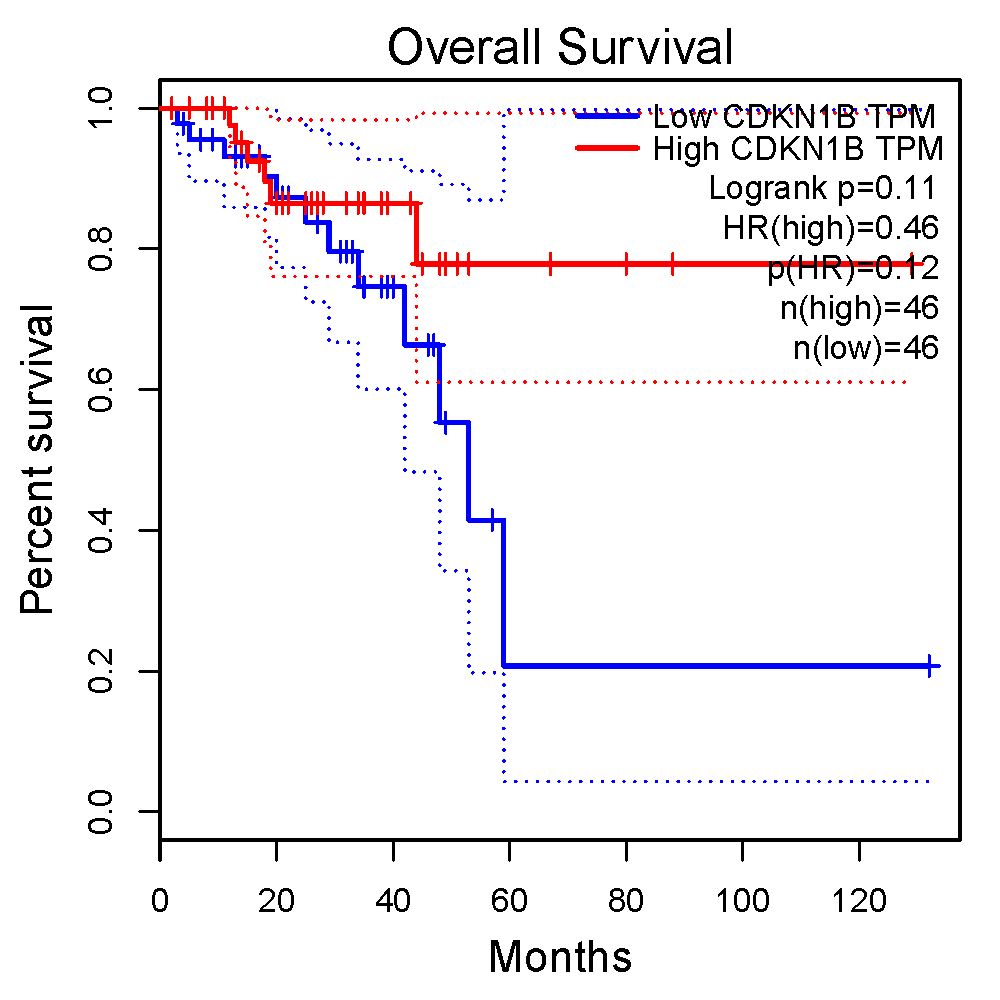 | 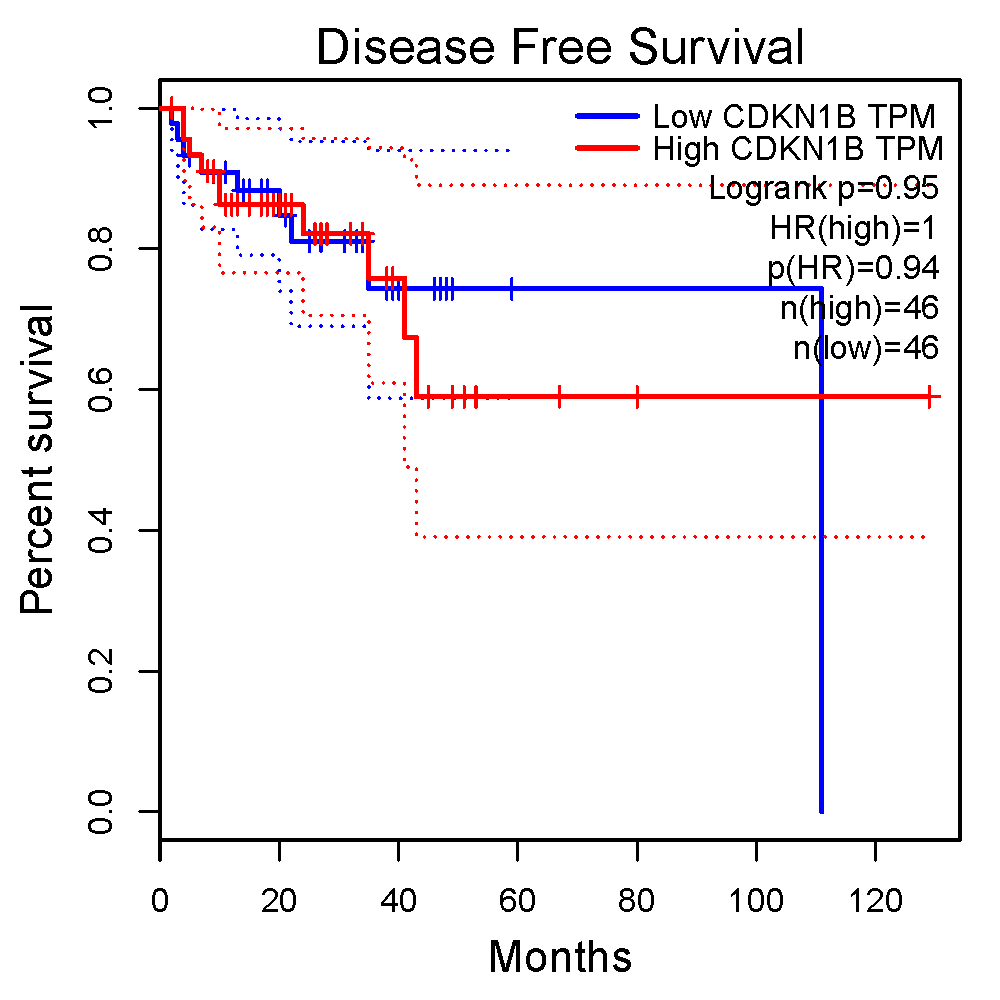 |

**Fig. S5**  Expression and clinical significance of CDKN1B mRNA in rectal cancer according to GEPIA: a. the expression difference of CDKN1B mRNA in cancer tissues and normal tissues; b. the association between CDKN1B mRNA expression and TNM staging; c. the association between CDKN1B mRNA expression and OS; d. the association between CDKN1B mRNA expression and DFS.

| a | b |
| --- | --- |
| 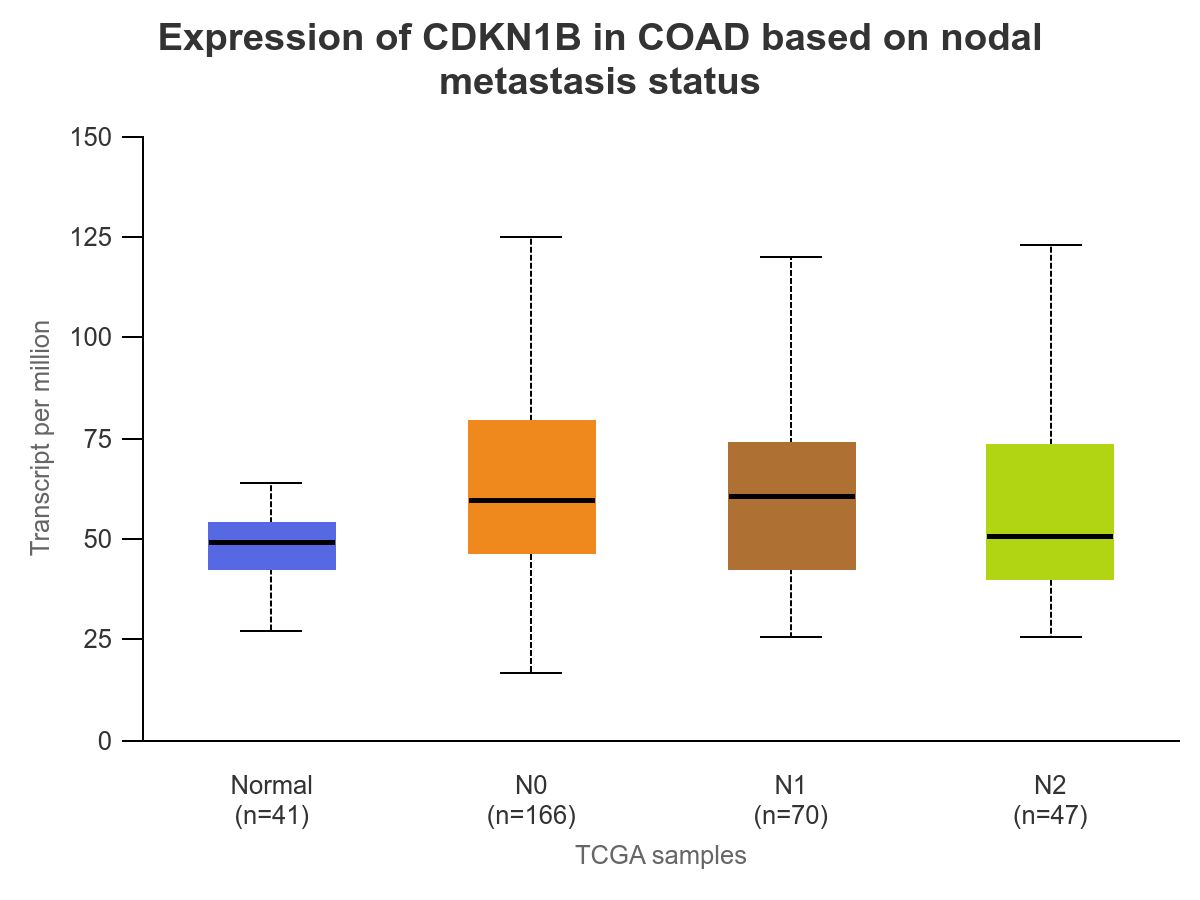 | 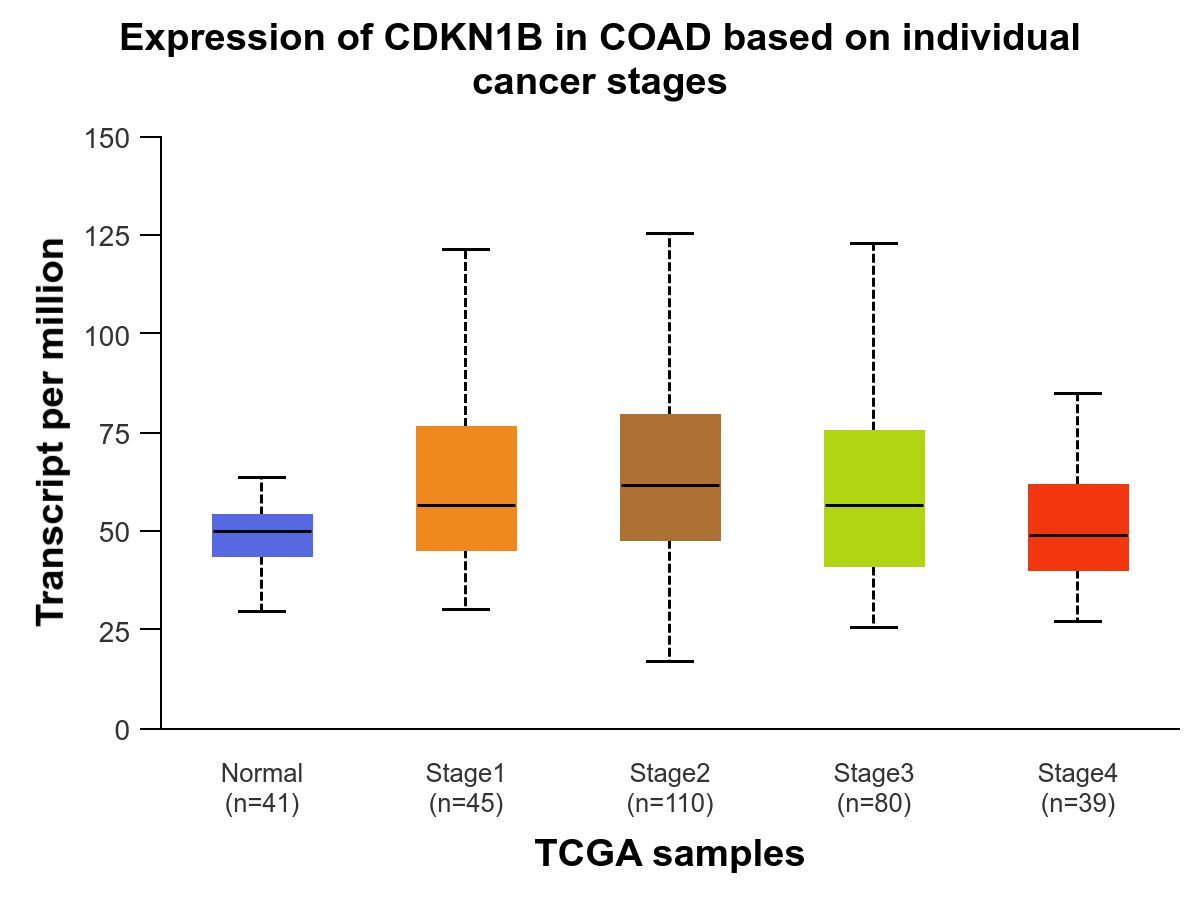 |
| c |  |
| 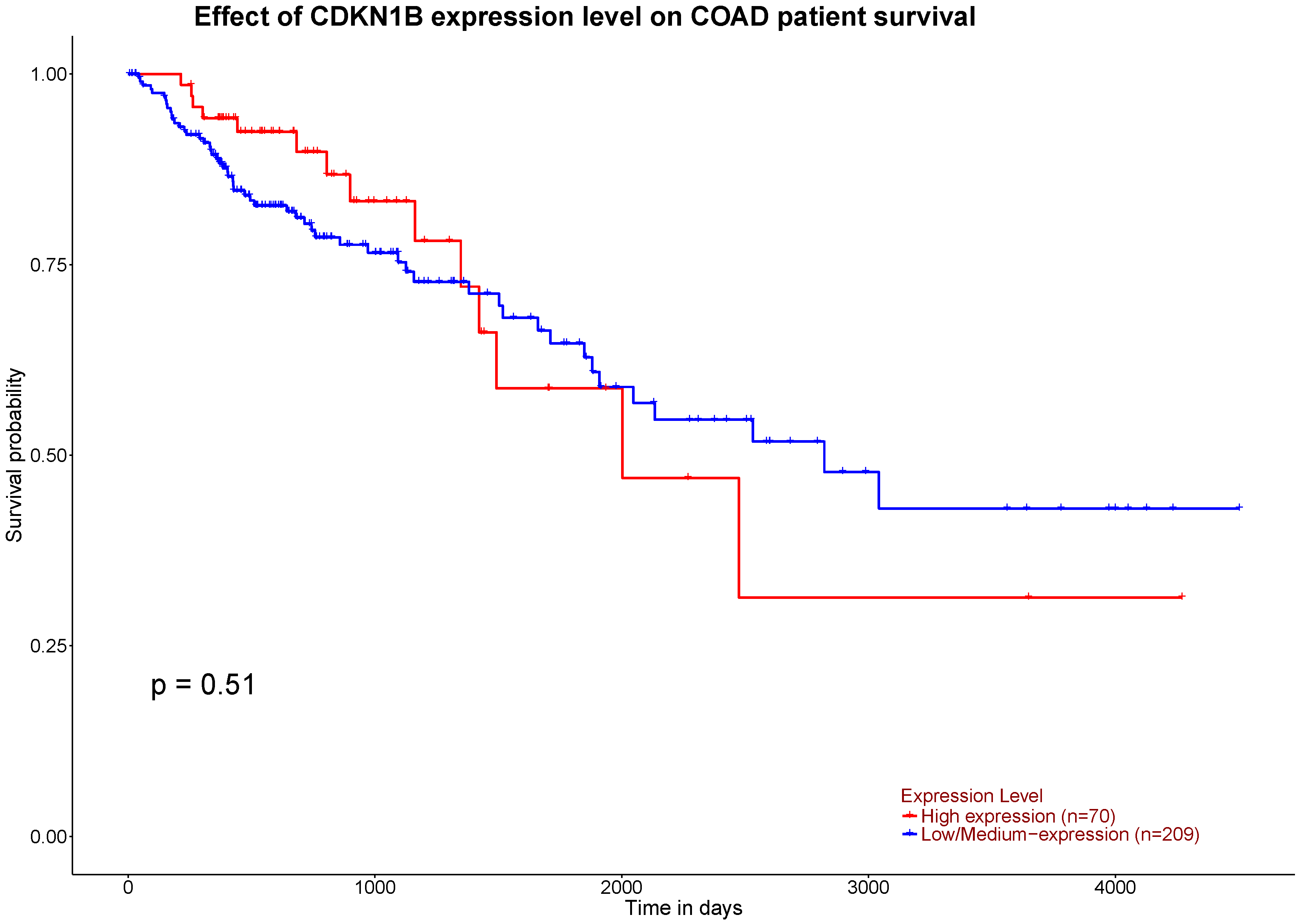 |  |

**Fig. S6**  Expression and clinical significance of CDKN1B mRNA in colon cancer according to UALCAN: a. the association between CDKN1B mRNA expression and lymph node metastasis; b. the association between CDKN1B mRNA expression and TNM staging; c. the association between CDKN1B mRNA expression and OS.

| a | b |
| --- | --- |
| 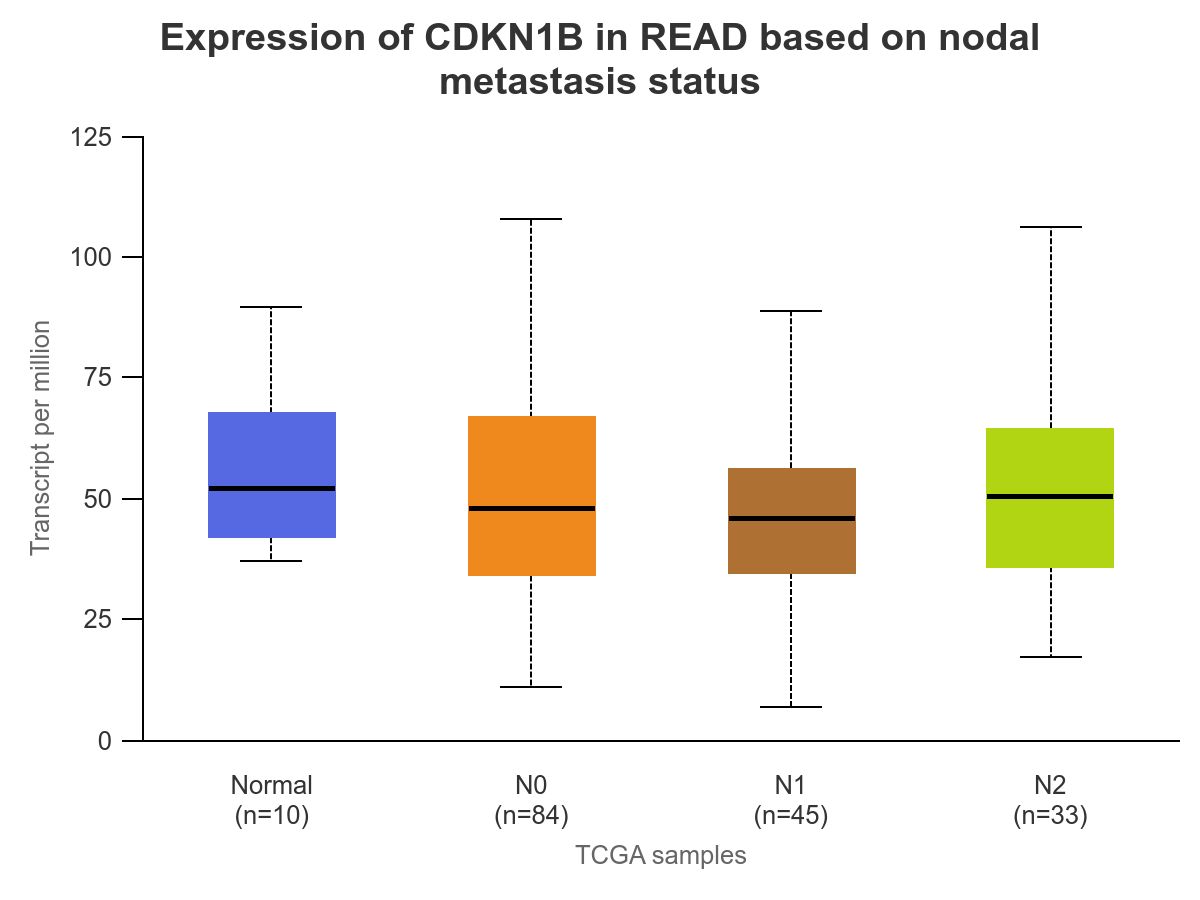 | 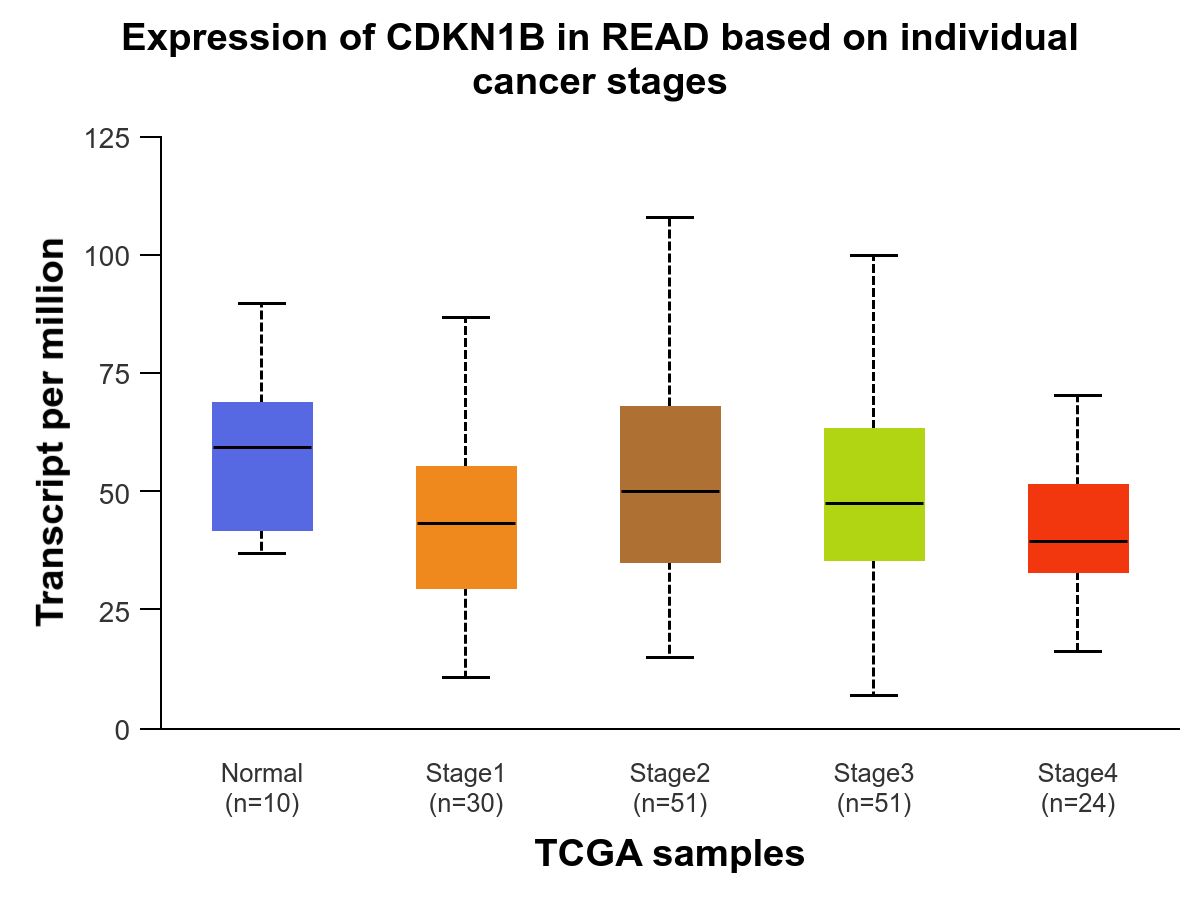 |
| c |  |
| 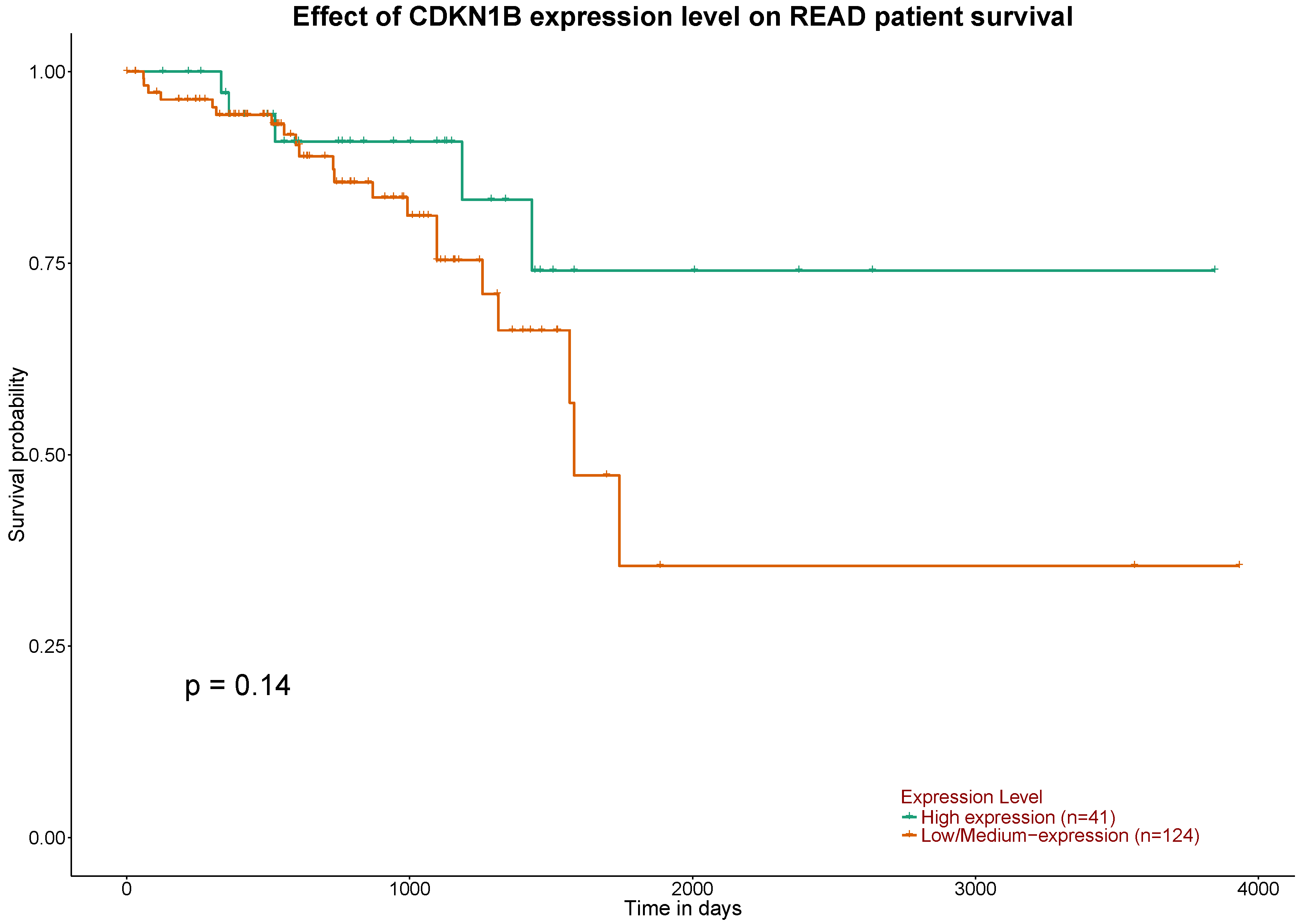 |  |

**Fig. S7**  Expression and clinical significance of CDKN1B mRNA in rectal cancer according to UALCAN: a. the association between CDKN1B mRNA expression and lymph node metastasis; b. the association between CDKN1B mRNA expression and TNM staging; c. the association between CDKN1B mRNA expression and OS.

1. **^*^ :**Correspondence:He Li [lihedoc@163.com](mailto:lihedoc@163.com)

   ^#^**:**Co-first author:Jing Zou, Dong Wang and Gaoping Yin

   1 Department of Radiology, Yantai Affiliated Hospital of Binzhou Medical University, 717 Jinbu Street, Yantai 264100, Shandong Province, China

   2 Department of Stomach and Intestine, Yantai Affiliated Hospital of Binzhou Medical University, 717 Jinbu Street, Yantai 264100, Shandong Province, China [↑](#footnote-ref-2)
